# Ectopic Pregnancy in the Round Ligament Following Bilateral Salpingectomy: A Case Report

**DOI:** 10.7759/cureus.47900

**Published:** 2023-10-29

**Authors:** Christian G Guevara, Zachary A Blashinsky, Isidro A Cardella

**Affiliations:** 1 Obstetrics and Gynecology, Florida International University, Herbert Wertheim College of Medicine, Miami, USA

**Keywords:** abdominal pregnancy, management, diagnostic challenges, salpingectomy, round ligament, ruptured ectopic pregnancy

## Abstract

Ectopic pregnancies, characterized by the implantation of a fertilized ovum outside the uterine cavity, typically occur in the fallopian tubes. However, rare cases have been reported where implantation occurs in atypical locations. Round ligament pregnancy, a rare form of ectopic pregnancy, poses significant risks and can lead to life-threatening complications. This case report describes the presentation and management of a 31-year-old gravida four, para two (G4P2012) female who presented with acute left lower quadrant and pelvic pain. The patient’s medical history included a prior bilateral salpingectomy. Physical examination revealed severe left lower quadrant tenderness with guarding. A positive urine pregnancy test and elevated serum quantitative beta-human chorionic gonadotrophin level of 1,735 mIU/mL (normal range: <5 mIU/mL) confirmed pregnancy. Transvaginal ultrasound revealed an empty intrauterine cavity with no gestational sac or fetal pole. A 2 cm cystic structure was identified attached to the left ovary. Ectopic pregnancy was diagnosed, methotrexate was administered, and the patient was discharged with a scheduled outpatient follow-up. However, she returned to the emergency room within 48 hours reporting persistent pelvic pain. At this moment, it was decided that emergent surgical intervention was required. The surgical exploration confirmed the presence of a ruptured ectopic pregnancy in the round ligament, requiring excision and hemostasis. This case report highlights the importance of considering abnormal localization of ectopic pregnancy as a differential diagnosis in women presenting with pelvic pain, even after bilateral salpingectomies. It emphasizes the challenges in diagnosis and management when ectopic pregnancy occurs in atypical sites and highlights the necessity for vigilant follow-up and prompt surgical intervention when medical management fails.

## Introduction

Ectopic pregnancies account for approximately 1-2% of all pregnancies and pose a significant risk to maternal health [1]. While the majority of ectopic pregnancies occur in the fallopian tubes, cases of implantation in other uncommon locations have been reported [2]. Abdominal ectopic pregnancies are exceptionally rare, at a rate of approximately 1% [3]. Abdominal ectopic pregnancy locations described in the literature have included the pelvic sidewall and broad ligament [3,4]. No cases of ectopic pregnancy in the round ligament have been previously reported in the literature. Ectopic pregnancies are the number one cause of first-trimester maternal morbidity [5]. Additionally, abdominal ectopic pregnancies have shown mortality rates as high as 7.7 times greater than tubal ectopic pregnancy [6]. This unique presentation emphasizes the need to consider abnormal localization to ensure proper diagnosis and consequential management to reduce complications. This case report describes an ectopic pregnancy in the round ligament of a patient who had previously undergone bilateral salpingectomy.

## Case presentation

A 31-year-old woman, gravida four, para two (G4P2012), presented to the emergency department with sudden-onset lower abdominal pain and vaginal bleeding. Her medical history was notable for a bilateral salpingectomy performed two years earlier for birth control during a cesarean section. The patient had a regular menstrual cycle, and her last menstrual period occurred two weeks before the presentation.

**Figure 1:**
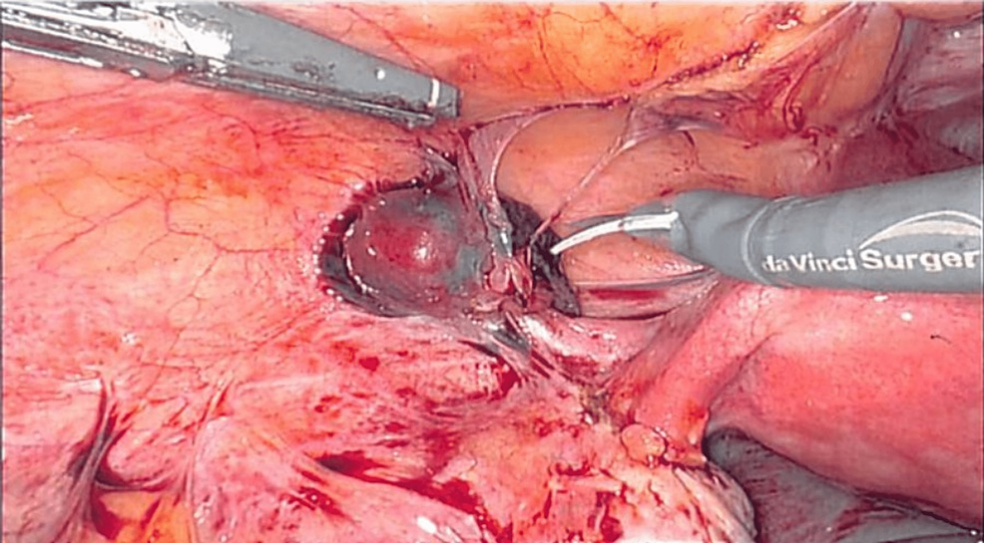
Dissection of ectopic pregnancy attached to the round ligament.

**Figure 2:**
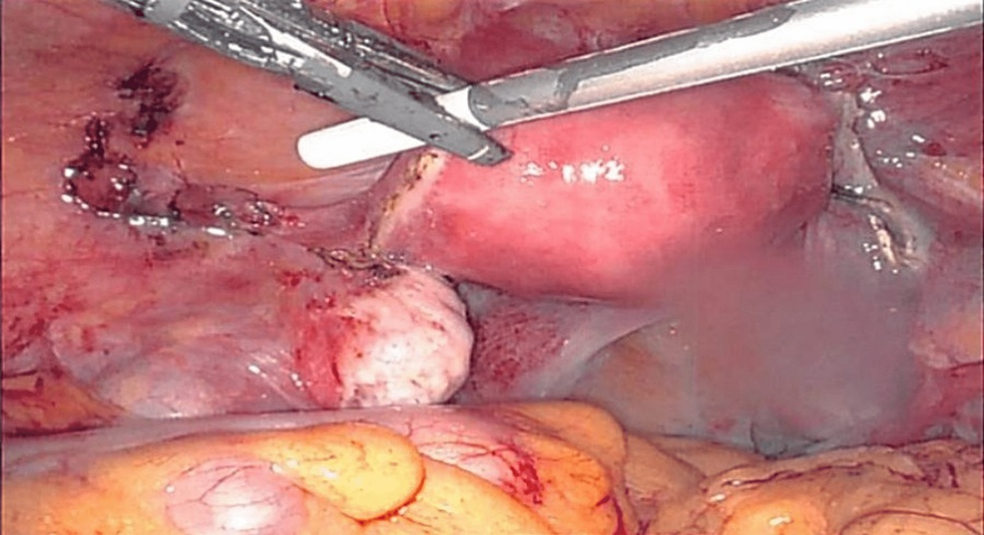
Removal of the cornual segment of the fallopian tube.

The patient appeared pale on physical examination and exhibited mild tenderness in the left lower quadrant. The patient had a positive pregnancy test and a beta-human chorionic gonadotropin (β-hCG) of 1,735 mIU/mL. A transvaginal ultrasound revealed an empty uterus with a complex adnexal mass on the left side. No intrauterine pregnancy was visualized. Considering the clinical presentation and imaging findings, an ectopic pregnancy was suspected. The patient was administered methotrexate and discharged with planned outpatient follow-up. However, the patient failed methotrexate treatment and returned to our institution’s emergency department 48 hours later, complaining of persistent, severe abdominal pain. At that point, a decision was made, and the patient underwent an exploratory laparoscopy, during which an ectopic pregnancy was identified within the round ligament on the left side. The operation was conducted robotically. The left ovary was observed and found to be clean with no evidence of ectopic. However, just above the left round ligament, a 2 cm ectopic was identified and dissected (Figure 1). Careful dissection of the round ligament facilitated the intact removal of the ectopic pregnancy. The tubal remnants were then removed in the cornual region of the tubes bilaterally (Figure 2). Severe pelvic adhesions were identified and properly lysed robotically. Hemostasis was achieved, and the round ligament was repaired. The postoperative course was uneventful, and the patient was discharged on postoperative day zero.

## Discussion

Here, we reported a unique case of an ectopic pregnancy in the round ligament, an exceptionally rare location of abdominal pregnancies. The exact mechanism leading to implantation in this location remains unclear. However, in cases where the fallopian tubes have been surgically removed, such as in our patient who had undergone bilateral salpingectomy, it is postulated that the fertilized ovum may reach the round ligament through retrograde migration or secondary implantation from the peritoneal cavity [7]. Other potential mechanisms of action include the development of micro-fistulas after salpingectomy or uterine perforation during embryo transfer in vitro fertilization [8]. Based on the patient history, it is likely the mechanism of implantation occurred as a result of retrograde migration. The diagnosis of ectopic pregnancy in the round ligament presents a challenge due to its rarity and atypical clinical presentation. The clinical manifestations often mimic those of tubal ectopic pregnancies, including abdominal pain and vaginal bleeding. Thus, a high index of suspicion is necessary, particularly in patients with a history of tubal surgery and a positive pregnancy test.

Transvaginal ultrasound is a valuable tool for diagnosing ectopic pregnancies, but its effectiveness may be limited in cases where the ectopic pregnancy is located outside the fallopian tubes [9]. In such instances, ultrasound findings may be less characteristic and inconclusive. Therefore, laparoscopy remains the gold standard for both the definitive diagnosis and treatment of ectopic pregnancies, enabling direct visualization and intervention.

Several cases of ectopic pregnancies in atypical locations following tubal surgery have been reported in the medical literature. In a systematic review by Yoder et al., 28 cases of abdominal or heterotopic ectopic pregnancies were identified. Overall, 50% of cases were identified to have received tubal surgery, with 32% specifically undergoing a bilateral salpingectomy [8]. Azhar et al. presented a ruptured ectopic pregnancy of the broad ligament that necessitated open laparotomy and hemostasis of the bleeding broad ligament and right pelvic sidewall [4]. This demonstrates the potential adverse outcomes if rapid diagnosis and treatment are not made. An ectopic pregnancy of the broad ligament differs from one in the round ligament such as our patient based on their differing anatomic structures and attachment sites. An ectopic pregnancy of the round ligament could theoretically present anywhere along the ligament from the superior uterus through the inguinal canal to its attachment to the labia majora.

These findings, as well as the case we describe here, suggest the importance of high clinical suspicion and rapid surgical management to preserve fertility and prevent complications in this cohort. Ultimately, management of ectopic pregnancy in the round ligament involves a surgical intervention to remove the ectopic pregnancy and achieve hemostasis. Surgical procedures in the pelvic space have several associated risks [10]. Transection of the round ligament may impact uterine support, and in patients wishing to maintain fertility, the utero-ovarian ligament, fallopian tubes, and cornua must be carefully avoided. Additionally, surgeons must properly identify the external iliac vessels, internal iliac artery, and ureter. While the decision to transect the round ligament for greater exposure is a clinical decision, evidence suggests that transection does not increase the incidence of dyspareunia, dysmenorrhea, chronic pelvic pain, or uterine prolapse [11]. Management of abdominal ectopic pregnancies varies based on length of gestation, location, and level of complication. A gravida one, para zero, with an 11-week abdominal pregnancy with a β-hCG of 53,680 mIU/mL was treated with bilateral uterine artery embolization and intramuscular methotrexate with complete resolution [12]. A seven-week ectopic located in the right posterior cul-de-sac that extended into the posterior vaginal wall was excised from the underlying peritoneum laparoscopically [13]. The use of reconstituted human thrombin with a gelatin, collagen, and cellulose matrix was used to manage excessive hemorrhage in a laparoscopic procedure to remove an abdominal ectopic pregnancy [14]. A previous case report by Noguchi et al. in Japan presented an ectopic pregnancy in a cyst of the canal of Nuck [15]. The canal of Nuck accompanies the round ligament through the inguinal canal to the labia majora. This patient underwent ultrasonography, magnetic resonance imaging, and dilation and curettage before an exploratory laparotomy was able to visualize and extract the mass. The variability in the location of abdominal ectopic pregnancies creates a challenge for formulating a clear and consistent management plan. While abdominal locations must remain on the differential for patients presenting with signs and symptoms of ectopic pregnancy without clear visualization on imaging, treatment decisions must be made by the physician, with surgical resection by laparotomy being the definitive choice.

## Conclusions

This case report presents a rare occurrence of ectopic pregnancy in the round ligament following bilateral salpingectomy. The diagnostic challenges associated with ectopic pregnancies in atypical locations underscore the importance of considering this possibility in patients with a history of tubal surgery who present with abdominal pain and vaginal bleeding. Laparoscopy remains the preferred approach for diagnosis and management. Further investigation into the etiology and pathophysiology of abdominal ectopic pregnancies is required. Understanding this unique mechanism of disease presentation can allow clinicians to develop guidelines for diagnosis and management. Increased awareness of this rare phenomenon is essential to ensure prompt intervention and prevent potentially severe complications.
